# National human papillomavirus vaccination (GH_PV) programme: Ghana at a crossroads; is vaccine supply alone enough to ensure success?

**DOI:** 10.3389/fgwh.2025.1667727

**Published:** 2025-09-15

**Authors:** Kwabena Amo-Antwi, Yvonne Nartey, Ramatu Agambire, Akwasi Antwi-Kusi

**Affiliations:** 1 School of Medical Sciences, Kwame Nkrumah University of Science & Technology/Komfo Anokye Teaching Hospital, Kumasi, Ghana; 2 Walter Aiden Specialist Clinic, Asokwa-Kumasi, Ghana; 3 Department of Adult Health, School of Nursing and Midwifery, University of Ghana, Accra, Ghana; 4 Department of Nursing, Garden City University College, Kumasi, Ghana

**Keywords:** cervical cancer, genital warts, nationwide human papillomavirus vaccination campaign, expanded programme on immunization, GH_PV programme

## 1 Introduction

Ghana stands at a critical point to include human papillomavirus (HPV) vaccination in the Expanded Programme on Immunisation (EPI) to combat cervical cancer. Cervical cancer is the second most common female cancer in Ghana (1). The role of high-risk human papillomavirus (hr-HPV), particularly HPV 16 and 18, in cervical carcinogenesis is firmly established by both local and international studies (2, 3). Annually, around 407 cervical cancer cases are managed at the two largest cancer treatment centres in the country, with a low overall 3-year survival rate of 39% (4, 5). In straightforward terms, if 10 cases are diagnosed today, only about 4 will survive for 3 years post-diagnosis.

The Ghana Health Service released a memorandum on June 2, 2025, to include the HPV vaccine in the routine immunisation schedules of all regions by September 2025. This commendable and timely project breaks new ground in the fight against cervical cancer; however, significant questions remain unanswered.

### 1.1 Health system readiness and capacity

Ghana, with its elaborate and well-structured health care system spanning from the Community-Based Health Planning and Services (CHPS) compounds to the referral tertiary institutions, has achieved an overall high vaccine access and utilisation, from the inception of the EPI programme to date. The EPI implements a standardised series of preparatory activities for the introduction of each new vaccine. This process encompasses meetings of the National Immunisation Technical Advisory Group (NITAG), planning sessions, budget formulation, training for health workers, communicators, and journalists, as well as the establishment of a monitoring and reporting system for both the campaign and routine immunisation. However, challenges persist regarding regional disparity in coverage, as seen in the current malaria vaccine rollout, which should inform critical appraisal of operational gaps and institution of solutions (6, 7). HPV vaccines must be stored and transported at temperatures ranging from 2 to 8 degrees Celsius across all 16 regions of Ghana. The existence of the three Walk-In Cold Rooms (WICRs), each with a capacity of 40 cubic metres, provides a net storage capacity of 28,571 L for vaccine preservation, thereby optimising storage for the national HPV vaccination programme. It is commendable that the 10 new 20 cubic-metre WICRs, six for newly established regions and four for contingencies, can maintain the chain up to the regional level. The receipt and handling of the vaccine at the facility level is the delicate end of the cold chain, which is of much concern. Although the country has received 567 Cold Chain Equipment (CCE) units from the Cold Chain Equipment Optimisation Platform 2, this is insufficient to meet the needs of approximately 2,000 health facilities at the district and facility levels, which lack cold chain equipment. While further support for CCE is anticipated from Africa Centres for Disease Control and Prevention (CDC) and other collaborators to address existing gaps, it is of paramount importance that regional, district, and facility heads of deprived sites should actively plan and coordinate regularly with nearby endowed health facilities for vaccine storage and transport.

In addition to the availability of the cold chain, it is essential to monitor the equipment for temperature incursions, which must be promptly identified and addressed to maintain vaccine efficacy. The cold chain temperature should be monitored at least twice a day, including weekends, alongside the continuous monitoring devices at the national level that offer 24 h temperature visibility within the WICR.

### 1.2 Target population and coverage strategy

The target age group aligns with World Health Organisation (WHO) recommendations, which focus on individuals aged 9–14 years (8). Per the compulsory basic education policy in Ghana, most of such girls will be in the upper primary and junior secondary school. The GH_PV programme roll-out is nationwide, ensuring coverage of out-of-school girls in the inner-city suburbs, including the “Kayayo herd porters” and daughters of pastoralist nomads, is paramount.

To promote an all-inclusive strategy for the vulnerable population, eligible girls in schools or communities receive vaccinations without inquiring about their HIV status. Targeting established specialised ART clinics nationwide will ensure that no eligible girl is overlooked, while also maintaining privacy and confidentiality. The existing EPI programme has faced challenges in reaching hard-to-reach populations, particularly island communities in the northern and southeastern regions of the Volta Basin in Ghana. These sites necessitate focused attention through the standard program, along with supplementary vaccination via targeted outreach initiatives to improve access and coverage.

### 1.3 Vaccine hesitancy and cultural sensitivity

Health provider communication (HPC) to parents and guardians is crucial in the uptake of the HPV vaccine (initiation, completion, and follow-through) (9). Other key persons/groups include adolescent groups, opinion leaders, and religious groups. The communication must identify guardian- or parent-specific hesitations and concerns, and address them promptly to enhance parental knowledge of the vaccine (10). Such communication must extend beyond vaccine safety and the prevention of cervical, vulvar and vaginal cancers to encompass vaccine efficacy against benign hyperproliferative epithelial lesions, including genital warts and recurrent respiratory papillomatosis, as well as cancers of the oropharynx, skin, and penis (11–13).

Adequate community education on the efficacy of the vaccine against these HPV-related diseases, presented in a clear and comprehensible manner, is essential for mitigating scepticism and addressing cultural sensitivities associated with the introduction of the vaccine. It is necessary to frame the HPV vaccine as a tool for protecting future mothers, not as linked to sexual behaviour. While the EPI communication arm, with its team of specialists, deserves applause for often crafting messages with appropriate content for previous exercises, similar attention is required for the GH_PV programme. Comprehensive sexual education of the adolescent and young individual has the potential to contribute significantly to the programme. The implementation of the programme can build on this critical phase in the Expanded Programme on Immunisation (EPI) by engaging teachers and educational sector leaders as strategic partners. Drawing on their pedagogical expertise and contextual understanding, these collaborations can facilitate the development and dissemination of culturally sensitive and socially acceptable messages aimed at increasing vaccine uptake among eligible school-aged girl.

### 1.4 Data recording and monitoring

Ensuring the collection and use of high-quality data during the HPV vaccine roll-out is critical to achieving coverage of at least 90% among eligible girls. Robust data systems will not only support effective monitoring and evaluation but also safeguard Ghana's progress towards the elimination of cervical cancer as a public health concern.

Both electronic and paper-based tools would be required to guarantee data completeness and accuracy. Past assessments of immunization data systems have identified challenges, including incomplete data and delays in reporting (14). In the context of the new campaign, it is advisable to hold onto vaccination registers, tally sheets, and summary reporting forms to collect case-based data. Furthermore, it is recommended that data aggregates be submitted to the national level through electronic platforms such as Google Sheets. To ensure data quality and accuracy, however, routine data recording and reporting tools may need to be revised and reformatted to integrate HPV vaccination indicators within the District Health Information System 2 (DHIS2) and Ghana's Health Management Information System (HMIS). This should include the capture of adverse events following immunisation (AEFIs), either electronically or through reporting to the nearest health facility.

Implementing a robust data system for real-time tracking of vaccine coverage and stock management is essential for program sustainability. A critical component of the program is the rapid response to events to alleviate concerns regarding vaccine hesitancy, which could adversely affect vaccine uptake. Designated personnel from the Food and Drugs Authority (FDA) and Ghana Health Service (GHS) should monitor all reported or suspected AEFIs, and the vaccine recipient must receive appropriate treatment. Given the susceptibility of data capture to human error, it is essential to approach data collection, capture and associated triangulations with particular attention to ensure the accuracy of the data.

As the programme advances, surveillance of HPV-infection-related endpoints can serve as a proxy estimate for assessing the early impact of the GH_PV vaccination programs. In the long term, data from existing population cancer registries will provide more reliable information on the burden of HPV-related conditions in the country over time hereby enabling continuous monitoring of progress towards cervical cancer elimination.

### 1.5 Sustainability and financing

The Government's decision to uncap monetary support from the 10% health sector allocation of National Health Insurance Levy Proceeds for the health budget, including immunisation, is timely. While initial funding from the Global Alliance for Vaccines and Immunisation (GAVI) and other partners is essential, mobilising domestic financing as the programme transitions in 5 years (2030) must not present a challenge if preparations are started in earnest.

Also, in our quest to sustain and fund the new vaccine programme, Public-Private Partnerships must be explored in time to enhance domestic revenue mobilisation efforts, to ensure the GH_PV programme beyond Gavi support. As Ghana transitions from Gavi support, Public–Private Partnerships (PPPs) should be actively explored as a mechanism for enhancing domestic revenue mobilisation and ensuring financial sustainability. Experiences from other low- and middle-income countries illustrate the potential of PPPs in strengthening immunisation systems. For instance, in Nigeria, collaborations with telecommunications companies have supported mobile technology for vaccine reminders and data reporting, while in Kenya and South Africa, partnerships with pharmaceutical companies and non-governmental organisations have facilitated HPV vaccine delivery in schools (15, 16). Leveraging private sector resources, innovation, and technical expertise could complement government efforts in Ghana, ensuring uninterrupted vaccine procurement, distribution, and delivery.

The EPI already provides routine immunisation services across the nation, effectively integrated into the primary healthcare system from the national to the community level. The intricate network of Community health nurses and officers will be evermore required to provide vaccination services using diverse strategies, including static points, mobile teams, and campouts in underserved areas. The Ministry of Health, as mandated by law, will have to diligently continue to recruit new staff to address gaps in the human workforce necessary for the nationwide delivery of immunisation services. The target population for the HPV vaccination must be accurately estimated periodically through data triangulation from multiple sources (Ghana Statistical Service, the Ministry of Education, and the United Nations) to enhance accuracy for adequate resource planning and deployment, thereby minimising stockouts and missed opportunities. Leveraging existing school health and adolescent programs may also offer opportunities to sustain the program.

### 1.6 Future considerations

Bivalent vaccines (containing types 16 and 18) have demonstrated significant cross-protection efficacy against cervical intraepithelial neoplasia (CIN) resulting from infections with types HPV 31 and HPV 33 (17). Notably, the subregion has a low presence of HPV 31, a vaccine-type hr-HPV, in women with cervical cancer (18–20). This supports the suggestion that HPV 31 infections rapidly clear after primary contact, reducing its carcinogenic potential (21). However, several sub-regional studies have documented significant levels of HPV 35 infections in women from a wide spectrum of the female population (18, 22–24). Likewise, non-vaccine low-risk types, HPV 43 and 44, featured more prominently than HPV 6 and 11. This knowledge of genotypic distribution in Ghana has implications for future vaccine choices and impact, as the protection afforded by the HPV vaccine is type-specific. Further studies on HPV 35, 43 and 44 strains for possible inclusion in future vaccines are a laudable step. Evidence supporting the effectiveness of a single-dose HPV vaccine is growing, and adopting this approach could improve coverage and reduce logistical burdens (25).

## 2 Conclusion

Introducing HPV vaccination in Ghana for all girls aged 9–14 years in all regions is a feasible and achievable goal. The healthcare provider's knowledge of HPV vaccination is central to the success of the GH_PV programme. However, it demands a well-coordinated, system-wide response, and strengthening the health system's readiness and capacity will ensure that Ghana can protect its girls and young women from preventable cervical cancer, contributing to better health outcomes and advancing gender equity in health.
